# Assessing one-shade composite resin color stability in response to everyday drinks

**DOI:** 10.1186/s12903-024-04612-z

**Published:** 2024-07-20

**Authors:** Mustafa Duzyol, Esra Duzyol, Burak Çarıkçıoğlu

**Affiliations:** 1 Faculty of Dentistry, Department of Restorative Dentistry, Istanbul Medeniyet University, Istanbul, Turkey; 2 Faculty of Dentistry, Department of Pediatric Dentistry, Istanbul Medeniyet University, Istanbul, Turkey; 3 Faculty of Dentistry, Department of Pediatric Dentistry, Çanakkale Onsekiz Mart University, Çanakkale, Turkey

**Keywords:** One shade, Color changes, Translucency, Contrast ratio

## Abstract

**Objective:**

The aim of our study was to measure the color changes in one-shade composite resins when exposed to common drinks, such as tea, cola and coffee.

**Materials/Methods:**

In our study, Omnichroma, Vitrra APS Unique, GC A’chord and Charisma Diamond One composite resins were used. Composite resins were placed in stainless steel molds with depths of 2 mm and diameters of 5 mm. Ten specimens were immersed in tea, 10 specimens were immersed in coffee, 10 specimens were immersed in cola and 10 specimens were immersed in distilled water in an incubator at 37 °C for 14 days. Color measurements were performed at the beginning of the study and after 24 h and 14 days. Color values ​​were measured using a CIE L*a*b* system with a spectrophotometer device. Color and translucency changes were calculated and data analyzed using one-way ANOVA, two-way ANOVA, and post-hoc Tukey test (*p* < 0.05).

**Results:**

The greatest color changes occurred in the tea and coffee groups; the smallest color change occurred in the control group. After 14 days, the greatest color change was observed in the Charisma + Coffee group; the smallest color change was observed in the Omnichroma + Water group. The transparency and contrast ratios changed in all groups, and the smallest change occurred in the Omnichroma control group.

**Conclusion:**

Significant differences were found in the composite color changes after immersion in beverages. The color variations significantly differed depending on the beverage in which the specimens were immersed. The initial contrast ratio was markedly different from the examined materials.

**Clinical significance:**

The study emphasizes the significance of common beverages on the color stability of one-shade composite resins, underlining the need of appropriate material selection for long-term aesthetic effects in one-shade composite resin restorations.

## Introduction

In the field of restorative dentistry, resin-based composites are quite common. An important component of the aesthetic satisfaction of patients in restorative treatment is the tooth structure and the structural and optical compatibilities of the composite restoration with adjacent teeth. Multilayer composites with different shades and opacities should be used together to craft the tooth appearance. Multilayer restorative treatment procedures require precise color determination and high clinical experience. This phenomenon complicates the treatment, increasing the dental chair time and cost. [1, 2] One-shade (monochromatic) composites aim to increase the efficiency while reducing the required technical precision, thus eliminating treatment complexity for clinicians. Unlike traditional methods relying on pigments, monochromatic composites offer a single-shade solution capable of adapting to various tooth shades. Manufacturers claim that these composites can replicate the color of adjacent teeth regardless of their original shade, a property referred to as the chameleon effect. [3]

Multilayer composites were previously used for this purpose, but they were limited in color range and required matching to specific VITA classic hue groups. Monochromatic composites, however, embrace a broader color matching principle, allowing them to blend seamlessly with all shades of teeth. By adopting monochromatic composite resins, dentists can achieve aesthetically pleasing results that merge flawlessly with natural dentition. This innovation represents a significant advancement in dental materials, promising improved outcomes for patients seeking dental restorations. [4]

Translucency is a main factor controlling aesthetics, and it is crucial for selecting composites. Translucency directly affects the lightness value, which is the most relevant aspect of color. [4] Translucency depends on the thickness of the material and the scattering and absorption coefficients of the pigments and opacifiers in the resin composite [5]. The color of the restorative material with the appropriate translucency closely matches the surroundings of the restoration and the structure of the teeth. The aesthetic results of composite restorations can be excellent with the right material selection and clinical experience.

Both internal and external factors can cause stain in the resin-based materials. The resin matrix composition, the filler loading amount, and the filler particle size are important intrinsic components. The type of staining agent, time of exposure and compatibility of the material with the resin matrix are extrinsic causes. [6] The color stabilities of composite resins are examined using an aging procedure or through immersion in different solutions, such as coffee, tea and red wine. The staining potentials of these solutions vary according to their compositions and properties. [7] Choosing a resin composite with an appropriate color, translucency and contrast ratio is essential for successful restoration. However, a main reason for replacing composite resin restorations is their discoloration in the oral environment. [5, 8, 9]

Therefore, the aim of this study is to evaluate the changes in the color, translucency and contrast ratio characteristics of four new generation resin composites after 24 h and 14 days of storage in water, coffee, tea and cola. The null hypotheses are as follows: (a) there should be no significant differences between the composites regarding changes in color, translucency and contrast ratio, and (b) there should be no significant differences between beverages for changes in color, translucency and contrast ratio.

## Materials and methods

### Specimen preparation

Sample size calculation was performed with the G*Power 3.1 software (Heinrich-Heine-Universität Düsseldorf, Düsseldorf, Germany) and the sample size was calculated as 10 per group with alpha-type error of 0.05, a power (1-beta) of 0.95, an effect size of 0.712 obtained from a previous study. [10] A total of 160 specimens (40 from each composite group) were prepared according to the manufacturer recommendations. Composite resins were placed in stainless steel molds with depths of 2 mm and diameters of 5 mm. Mylar strips and glass slides were placed on each specimen to prevent air bubbles, and pressure was applied. Thus, a smooth surface was obtained by removing excess material. Then, the specimens were polymerized for 20 s using a light emitting diode (LED) source (Elipar Freelight II, 3 M ESPE, AG, Germany, 1150 mW/cm^2^) and removed from the molds. Before polymerization in every five specimens, the power of the light source was checked; it was ensured that the power was higher than 1000 mW/cm^2^. Standardization was achieved by measuring the thicknesses of the specimens with a digital caliper (Ultra-Cal V, Fowler Corp., Sylvac, Switzerland). After polymerization, the specimens were polished with aluminum oxide-coated discs (Sof-Lex™ XT; 3 M/ESPE, St. Paul, MN, USA). The polishing and numbering processes of the specimens in each composite group were performed by the same investigator (BC).Table 1

**Table 1:** Materials used in the study

Composite Resin	Composition^a^		Inorganic Particle Size	Manufacturer
	Organic Matrix	Inorganic Matrix (% by Weight)		
**Vittra APS Unique**	TEGDMA and UDMA	Nanospheres of a zirconia complex, 200 nm; (72%)	Nanoparticulate	FGM, Joinville, Santa Catarina, Brazil
**GC A’chord**	BisMEPP, TEGDMA, UDMA	Silicon dioxide (silica), stabilizers and pigments; (68%)	Nano Hybrid	GC Europe, Leuven, Belgium
**Omnichroma**	TEGDMA, UDMA	Uniformly sized suprananospherical particles (260-nm spherical SiO_2_–ZrO_2_); (79%)	Nanofilled Composite	Tokuyama Dental Corporation Tokyo, Japan
**Charisma Diamond One**	TCD-DI-HEA, UDMA	Ba–Al–F Borosilicate glass, prepolymerized SiO_2_ nanofiller; (81%)	Nano Hybrid	Kulzer, Germany

TEGDMA, triethylene glycol dimethacrylate; UDMA, urethane dimethacrylate; BisMEPP, 2,2’-bis-(4-methacryloylethoxyphenyl) propane; TCD-DI-HEA, bis-(acryloyloxymethyl) tricycle-[5.2.1.02.6] decane; SiO_2_, silicon dioxide; ZrO_2_, zirconium dioxide; Ba, barium; Al, Aluminium; F, Fluor

^**a**^Data from manufacturers

### Color measurements

In this study, color values were measured using a Commission International de l’Eclairage (CIE) L*a*b* system with a spectrophotometer device (VitaEasyshade V, Vita Zahnfabrik, Bad Sackingen, Germany) using a neutral gray background (L*=64.1; a*=0.3; b*=−3.4). Measurements were performed under D65 standard lighting conditions, and the spectrophotometer was calibrated according to the manufacturer recommendations before each measurement. After the specimens were dried with tissue paper, three measurements were repeated on each surface; the average L, a and b values were calculated. The principle of the CIELAB system is based on the sensitivities of three types of conical adherent sensing cells to red, blue and green light. On this basis, each color would be represented by the abbreviations L, a, and b. The L* value (Lightness) would indicate the lightness and darkness of the color, ranging from 0 to 100, while a* (red to green) and b* (blue to yellow) would indicate the hue. [11]

Initial color measurements (L_0_, a_0_, b_0_) of the specimens were made after storage in distilled water at 37 °C for 24 h. The specimens were divided into 4 different groups according to the type of composite resin; after the first color measurement, they were randomly divided into 4 different subgroups (*n* = 10). Ten randomly selected specimens were immersed in tea (Lipton Yellow Label Tea, Unilever, Istanbul, Turkey), 10 specimens were immersed in coffee (Nescafe Classic, Nestle, Switzerland), and 10 specimens were immersed in cola (The Coca-Cola Company, Istanbul, Turkey). As a control group, 10 specimens were immersed in distilled water in an incubator at 37 °C for 14 days. The solutions were prepared and refreshed daily. In our study, tea was prepared by immersing 1 tea bag recommended for a standard cup size in 250 mL of boiling water. Coffee was prepared by dissolving 3.6 g of coffee in 300 mL of boiling water and by mixing for 10 min. A new cola bottle was used every day. The specimens extracted from the solutions were washed in distilled water for 5 min and dried. The color measurements were performed at the end of the 1st and 14th days as previously explained.

### Color, translucency and contrast ratio measurements

The color change levels in the specimens were calculated using the following CIEDE2000 formulation: [12]

$$\eqalign{\Delta {E_{00}} = & {\left[ {\left( {{{\Delta {L^{'}}} \over {{K_L}{S_L}}}} \right)} \right.^{2}} + {\left( {{{\Delta {C^{'}}} \over {{K_C}{S_C}}}} \right)^{2}} + {\left( {{{\Delta {H^{'}}} \over {{K_H}{S_H}}}} \right)^{2}} \cr \\&{\left. { + {{\rm{R}}_T}\left( {{{\Delta {C^{'}}} \over {{K_C}{S_C}}}} \right)\left( {{{\Delta {H^{'}}} \over {{K_H}{S_H}}}} \right)} \right]^{{1 \over 2}}} \cr}$$

The *ΔL’*, *ΔC’* chroma, and *ΔH’* color hue values are included in this formula. The R_T_ rotation function is the general rotation function that explains the interactions between chroma and hue differences in the blue region. The weighting function (*S*_*L*_, *S*_*C*_, and *S*_*H*_) adjusted for variations between the total color differences of pairs at coordinates L*, a* and b*. *K*_*L*_, *K*_*C*_ and *K*_*H*_ were the accurate terms for the test conditions that were set to 1. [13]

$${\text{RT}}{{\text{P}}_{{\text{TP}}_{00}}} = \sqrt {\begin{aligned}& {\left( {\frac{{{L^{'}}_B - {L^{'}}_W}}{{{K_L}{S_L}}}} \right)^2} + {\left( {\frac{{{C^{'}}_B - {C^{'}}_W}}{{{K_C}{S_C}}}} \right)^2} + {\left( {\frac{{{H^{'}}_B - {H^{'}}_W}}{{{K_H}{S_H}}}} \right)^2} \\ & + {{\text{R}}_T}\left( {\frac{{{C^{'}}_B - {C^{'}}_W}}{{{K_C}{S_C}}}} \right)\left( {\frac{{{H^{'}}_B - {H^{'}}_W}}{{{K_H}{S_H}}}} \right) \\ \end{aligned}}$$

The ΔE_00_ value was 0.8, and the threshold values were taken as references for perceptibility (PT); the 1.8 threshold values were taken as references for acceptability (AT).

Translucency parameters (TPs) and contrast ratios (CRs) were used to evaluate changes in translucency. TP was determined by calculating the L*, a*, b* values recorded on white (W) and black (B) backgrounds according to the following formula. [14]

CR was calculated as the light reflection ratio of the specimens on black (YB) and white backgrounds (YW) using the following formula: [15]

$$CR\, = \,\left( {{{{Y_B}} \over {{Y_W}}}} \right)$$

Differences in the TP and CR values were calculated using the following formula:

ΔTP = TP_after staining_ − TP_baseline_.

ΔCR = CR_after staining_ − CR_baseline_.

### Statistical analysis

IBM SPSS software (IBM Corp. Released 2019. IBM SPSS Statistics for Windows, Version 26.0. Armonk, NY, USA) was used for statistical analysis. Changes in ΔE_00_ values at 4 h and 14 days, as well as Contrast Ratio changes (ΔCR) and Translucency changes (ΔTP) values of resin composites and solutions, were compared using one-way ANOVA. Post-hoc Tukey test was used to make multiple comparisons. Changes in ΔE_00_, ΔCR and ΔTP values of the specimens in the same solution according to time were analyzed by paired-samples T test. A p-value of less than 0.05 was considered statistically significant.

## Results

The 24-hour and 14-day color change mean (ΔE_00_) and standard deviation (SD) values of all specimens in different solutions are presented in Table 2.

When 24-hour and 14-day immersion periods were compared in terms of ΔE_00_, specimens immersed in water showed the lowest values among all tested materials, and coffee showed the highest values.

**Table 2:** Mean ΔE_00_ values ± standard deviations of composites after 24 h and after 14 days of immersion in solutions

	Charisma	Omnichroma	Vittra	Achord
**ΔE**_**00**_ **24 h**				
Tea	3,8 ± 0,6aA	2,3 ± 0,8aB	3,6 ± 0,8aA	1,2 ± 0,4bC
Coffee	3,9 ± 0,8aA	2,3 ± 0,5aB	3,3 ± 0,5aA	1,8 ± 0,3aB
Cola	1,2 ± 0,6bAB	0,8 ± 0,3bAB	1,3 ± 0,4bA	0,7 ± 0,7bcB
Water	0,5 ± 0,1cAB	0,4 ± 0,1bB	0,6 ± 0,2cA	0,4 ± 0,1cB
Δ**E**_**00**_**14d**				
Tea	5,6 ± 0,9aA	3,4 ± 0,9aB	5,0 ± 0,6aA	1,6 ± 0,5bC
Coffee	6,1 ± 0,9aA	3,7 ± 0,6aC	5,1 ± 0,5aB	3,1 ± 0,6aC
Cola	2,6 ± 0,7bA	2,2 ± 0,4bA	2,0 ± 0,4bA	2,6 ± 0,8aA
Water	1,0 ± 0,2cA	1,0 ± 0,1cA	1,1 ± 0,3cA	1,2 ± 0,4bA

Lowercase letters indicate differences in the column, uppercase letters indicate differences in the rows

**Fig. 1:**
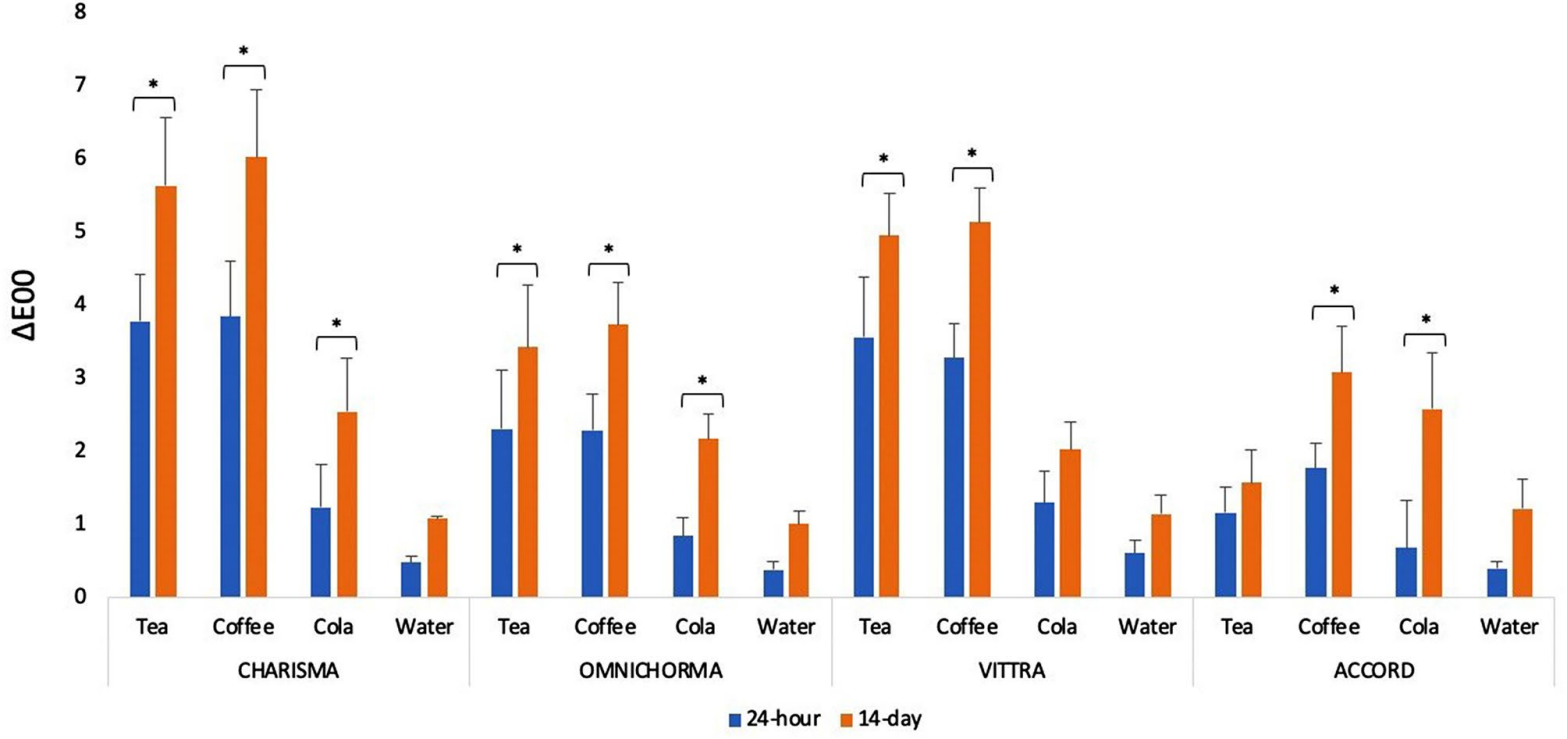
Time-dependent changes in ΔE_00_ values taken from 2 measurements

When the 24-hour and 14-day immersion periods were compared, there were no significant differences in all tested materials in water-immersed specimens; those immersed in coffee had significantly higher ΔE_00_ values (Fig. 1).

“*” indicates a statistically significant difference with time in the same group. Green horizontal line at 0.8 (ΔE_00_ units) represent the thresholds for perceptible (PT) and red horizontal line at 1.8 (ΔE_00_ units) represent the thresholds for acceptable (AT) values.

Translucency changes (ΔTP) of all specimens in different solutions after 14 days are presented in Table 3.

**Table 3:** Mean ΔTP values and standard deviations of composites after 14 days of immersion in solutions

ΔTP	CHARISMA	OMNICHROMA	VITTRA	ACCORD
**Tea**	-1,8 ± 1,5Aa	-1,7 ± 0,5Ac	-1,3 ± 0,9Bg	-1,1 ± 0,9Bi
**Coffee**	-1,5 ± 1,1Ca	-0,6 ± 0,5Dd	-0,6 ± 0,6Dh	-1,1 ± 0,5Ci
**Cola**	-1,7 ± 1Ea	-1,8 ± 1,2Ec	-0,9 ± 1,4Fh	-0,6 ± 0,5Fj
**Water**	-0,7 ± 0,4Gb	-0,2 ± 0,2He	-0,5 ± 0,5Gh	-0,5 ± 0,2Gj

Lowercase letters indicate differences in the column, uppercase letters indicate differences in the rows

Contrast ratio changes (ΔCR) of all specimens in different solutions after 14 days are presented in Table 4.

**Table 4:** Mean ΔCR values and standard deviations of composites after 14 days of immersion in solutions

ΔCR	CHARISMA	OMNICHROMA	VITTRA	ACCORD
**Tea**	0,051 ± 0,035Aa	0,041 ± 0,012Ad	0,042 ± 0,028Ag	0,026 ± 0,026Bi
**Coffee**	0,037 ± 0,028Cb	0,019 ± 0,014De	0,018 ± 0,019Dh	0,028 ± 0,013Ci
**Cola**	0,044 ± 0,026Da	0,041 ± 0,034Dd	0,028 ± 0,04Eg	0,018 ± 0,013Ej
**Water**	0,016 ± 0,011Fc	0,002 ± 0,004Gf	0,016 ± 0,015Fh	0,014 ± 0,006Fj

Lowercase letters indicate differences in the column, uppercase letters indicate differences in the rows

**Fig. 2:**
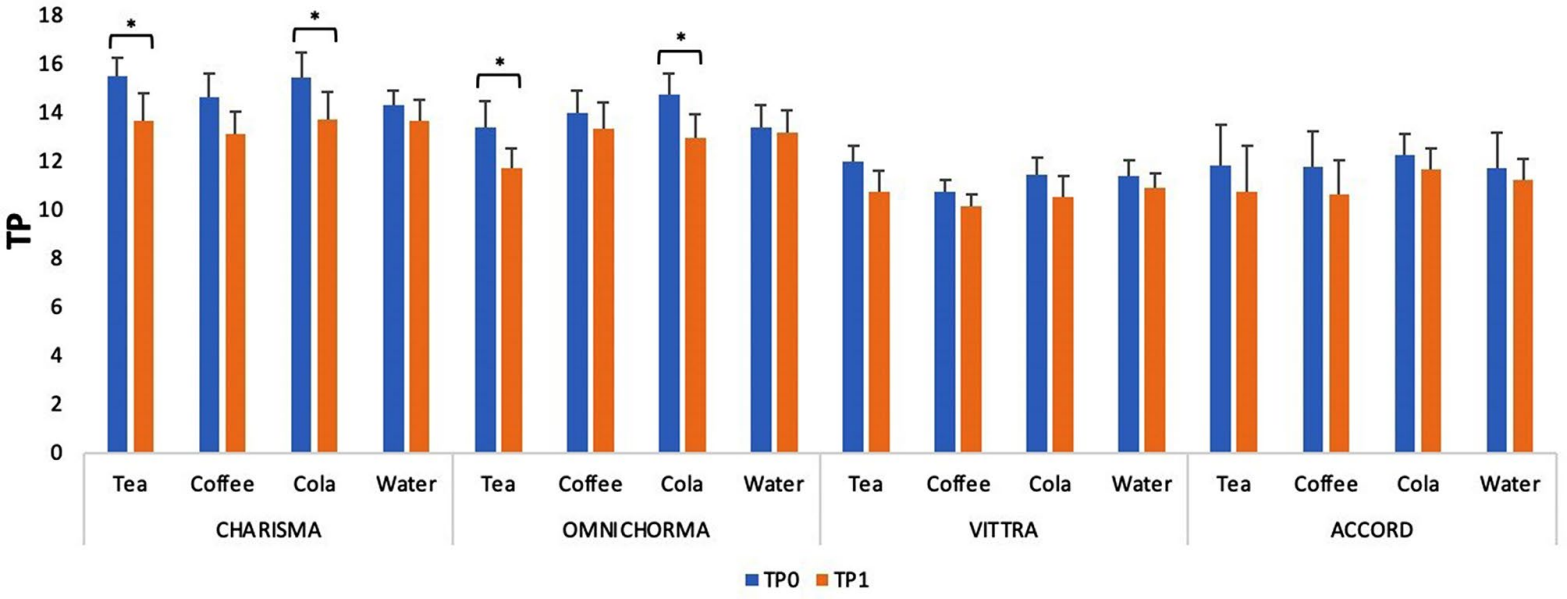
Time-dependent changes in TP (translucency parameters) values taken from 2 measurements “*” indicates a statistically significant difference with time in the same group

According to Fig. 2, there was a statistically significant decrease in translucency in the Charisma and Omnichroma specimens immersed in tea and cola. No statistically significant change was observed in the other groups.

**Fig. 3:**
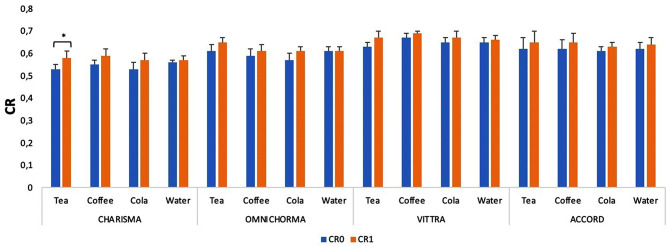
Time-dependent changes in CR (contrast ratio) values taken from 2 measurements. “*” indicates a statistically significant difference with time in the same group

According to Fig. 3, when changes in the CR values were evaluated over time, opacity increased significantly only in the Charisma specimens immersed in tea.

## Discussion

Discoloration is a major cause of aesthetic failure of restorations; it is often the main reason for replacing composite resin restorations. [16] In many studies, color changes in aesthetic restorative materials that are thought to be caused by beverages, such as tea, coffee and cola, have been investigated. [17] This study examined the effects of different beverages on single-color composite resins, it was hypothesized that there would be no significant differences between the groups regarding changes in color, translucency, and contrast ratio. Consequently, all hypotheses were rejected. Contrary to the null hypothesis (a), which posited no significant differences between the composites regarding these properties, the results revealed notable variations in color stability and optical characteristics, with Charisma and Vittra showed the greatest color change among all beverage groups. Similarly, hypothesis (b), suggesting no significant differences between beverages for changes in color, translucency, and contrast ratio, was refuted by the observed differences in staining propensity and reduction in translucency when immersed in tea or coffee compared to cola.

In this study, the color and translucency changes and contrast ratios of one-shade composites were analyzed after immersion in tea, coffee, cola and distilled water for 24 h and 14 days. The color differences in this study were reported on a neutral gray background. Absolute color coordinates (L, a, and b) allowed the color difference (ΔE) to be compared numerically. [18] The visual thresholds of perceptibility and acceptability describe the agreements in the color, translucency and whiteness in dentistry. These thresholds are of great importance as guidelines for restorative material selection, clinical performance evaluation, and restorative dentistry standardization. [19] In our study, ΔE_00_ values of 0.8 for perceptibility and 1.8 for acceptability were selected as references. However, there was no consensus on the acceptable amount of color difference. [20]. Mokrzycki and Tatol [21] organized the color changes in different values: 0 < ΔE < 1 indicated that change was not noticeable; 1 < ΔE < 2 indicated that only an experienced observer could notice the change; 2 < ΔE < 3.5 indicated that the change could be noticed by an inexperienced observer; 3.5 < ΔE < 5 indicated a significant color change; and 5 < ΔE indicated changes that were perceived as two different colors rather than discolorations of the original color.

Specimens were immersed in beverages for 24 h and 14 days. The color changes in the composite materials were significantly different between beverages. At the end of 24 h and 14 days, in specimens immersed in tea showed the highest ΔE_00_ value in Charisma and Vittra, while it showed the lowest ΔE_00_ value in Achord. In specimens immersed in coffee, Charisma and Vittra showed the highest ΔE_00_ values, while Accord and Omnichroma showed lower ΔE_00_ values. No statistical difference was observed in ΔE_00_ values after 14 days in all composites immersed in cola (Table 2). When 24-hour and 14-day immersion periods were compared in terms of Δ*E*_00_ value, coffee showed statistical differences in all composites. While tea and cola showed statistical differences in Charisma and Omnichroma; Tea in Vittra and cola in A’ccord showed a statistically significant difference (Fig. 1). As shown in previous studies, the color changes in composite resins immersed in water were determined to be not noticeable in our study. [6, 22] Values of ΔE_00_ at or below the PT threshold indicate an excellent color match. If the color difference is between the PT and AT thresholds, it signifies an acceptable change in color; values exceeding the AT threshold denote an unacceptable change in color. [23] After 24 h and 14 days; the ΔE_00_ values of all specimens immersed in coffee and all specimens except A’chord immersed in tea were above AT. ΔE_00_ values of all specimens immersed in cola were between AT and PT after 24 h and above AT after 14 days. ΔE_00_ values of all specimens immersed in water were below PT after 24 h and between PT and AT after 14 days (Table 2). In this study, tea and coffee, which have more dark or yellow colorants, caused more discoloration than the other solutions; these findings were in agreement with other studies in the literature. [24, 25]

The resin matrix content significantly affected the color change of the composite resin. Sensi, et al. [26] showed that water uptake and stain susceptibility decreased according to triethyleneglycol dimethacrylate (TEG–DMA) content. Bis-GMA is viscous; thus, composite resin restorative materials would require the addition of low molecular weight monomers to increase their consistency. In composite resins, the TEG–DMA molecule, which has low viscosity and excellent copolymerization properties, is frequently used as a diluent monomer for Bis-GMA. [22] Composite resins contain urethane dimethacrylate (UDMA) monomers, which have low water absorption and solubility properties and are more resistant to staining than Bis-GMA. In this study, G-aenial A’chord and Omnichroma, which were kept in various beverages, underwent less color changes than all of the solutions except for coffee. Pedrosa et al. [27] stated that advanced polymerization system (APS) technology used in Vittra Unique changes the color less than other resin matrix systems. However, this result was not obtained in our study. However, Vittra Unique showed the smallest color change in cola with high acidity levels.

A primary factor in evaluating the aesthetics of tooth color restoration procedures is the translucency value; the CIEDE2000 color difference formula is recommended for its calculation. [14] In this study, the TP00 formula was used to evaluate the data on translucency differences. Recent studies showed that the translucency of the restorative material varied with thickness. In this study, the specimen thickness was determined to be 2 mm, which was in agreement with recent studies. [2, 14] In their study by Lucena et al., [2] Omnichroma showed the largest translucency values ​​for all thicknesses. In our study, a statistically significant change in translucency was observed in the groups Omnichroma immersed in tea and cola. Previous studies reported a higher translucency of Bis-GMA-based resins than UDMA/TEGDMA-based resins because Bis-GMA has a refractive index closer to that of a silica filler than TEGDMA [2, 28]. Researchers found a negative relationship between the filler content and the TP value when the filler size remained unchanged. [2] With limited content information provided by manufacturers, Omnichroma with a UDMA and TEDGMA matrix and 260-nm suprananospherical fillers was the most translucent material among the specimens. The amount and shape of the filler potentially caused this result. According to Fig. 2, only tea and cola statistically affected the translucency over time in Charisma and Omnichroma specimens, while beverages did not statistically affect the translucency values in Vittra and Accord. Coffee did not statistically affect translucency in any specimen.

Differences in material composition can cause differences in color and contrast ratios. Although no precise information exists on the opacity levels of resin composites, this level is used to mimic the translucency characteristics of enamel and dentin. [29]. The enamel and dentin contrast ratio translucency levels can be thought of as inverses of each other [4]. In this sense, a material with a high contrast ratio has a low translucency and can be considered opaque. [30]. Vattanaseangsiri et al. [3] stated that the translucency of composite resins is more affected by the total amount of fillers than by particle size. Similarly, in our study, the CR0 values of Charisma, which has more filler content, were found to be lower. (Fig. 3). But in this study, when the 24-hour and 14-day immersion periods were compared in terms of CR, it was seen that the beverages had no statistical effect in the composite specimens, only the Charisma had a statistical effect in the tea.

The long-term effects of the results obtained in this study should be supported by investigating in vitro studies. Additionally, in vivo studies should be planned to evaluate color changes in the oral environment. More comprehensive research can be conducted on the effect of color change in composite resin materials with different monomer contents. Considering all these conditions, a broader experimental approach is needed for a comprehensive evaluation of color and contrast change.

## Conclusion

Within the limits of this study, significant differences were found in the color changes in the composites after immersion in beverages. Tea and coffee composites stained to a higher degree than Cola. Clinically detectable color change was observed in all materials when immersed in tea or coffee. After 14 days, the greatest color change was seen in Charisma for all beverages. Additionally, the color variations significantly differed depending on the beverage in which the ingredients were immersed. Translucency was significantly reduced in groups where Omnichroma and Charisma composite resins were immersed in tea or coffee. Among the materials examined, the contrast ratio was significantly different only in the Charisma immersed in tea.

## Data Availability

The datasets used and/or analysed during the current study available from the corresponding author on reasonable request.
